# High blood pressure predicts hippocampal atrophy rate in cognitively impaired elders

**DOI:** 10.1002/dad2.12035

**Published:** 2020-05-17

**Authors:** Cassidy M. Fiford, Jennifer M. Nicholas, Geert Jan Biessels, Christopher A. Lane, M. Jorge Cardoso, Josephine Barnes

**Affiliations:** ^1^ Department of Neurodegenerative Disease, Dementia Research Centre UCL Institute of Neurology London UK; ^2^ London School of Hygiene and Tropical Medicine London UK; ^3^ Department of Neurology and Neurosurgery Brain Center Rudolf Magnus University Medical Center Utrecht the Netherlands; ^4^ School of Biomedical Engineering and Imaging Sciences King's College London London UK; ^5^ Department of Medical Physics and Biomedical Engineering University College London London UK

**Keywords:** Alzheimer's disease, blood pressure, cognitive decline, hypertension, hypotension, longitudinal, mild cognitive impairment

## Abstract

**Introduction:**

Understanding relationships among blood pressure (BP), cognition, and brain volume could inform Alzheimer's disease (AD) management.

**Methods:**

We investigated Alzheimer's Disease Neuroimaging Initiative (ADNI) participants: 200 controls, 346 mild cognitive impairment (MCI), and 154 AD. National Alzheimer's Co‐ordinating Center (NACC) participants were separately analyzed: 1098 controls, 2297 MCI, and 4845 AD. Relationships between cognition and BP were assessed in both cohorts and BP and atrophy rates in ADNI. Multivariate mixed linear‐regression models were fitted with joint outcomes of BP (systolic, diastolic, and pulse pressure), cognition (Mini‐Mental State Examination, Logical Memory, and Digit Symbol) and atrophy rate (whole‐brain, hippocampus).

**Results:**

ADNI MCI and AD patients with greater baseline systolic BP had higher hippocampal atrophy rates ([r, *P* value]; 0.2, 0.005 and 0.2, 0.04, respectively). NACC AD patients with lower systolic BP had lower cognitive scores (0.1, 0.0003).

**Discussion:**

Higher late‐life BP may be associated with faster decline in cognitively impaired elders.

## 1 BACKGROUND

Hypertension is related to cognitive decline and dementia,^1^ including Alzheimer's disease (AD).^2^, ^3^ The link between hypertension and brain tissue loss (atrophy rate), is poorly understood, with conflicting results.^4^, ^5^, ^6^, ^7^

Hypertension may be a modifiable risk factor not just for prevention, but also progression of AD.^8^ The rate of progression in AD is heterogeneous and blood pressure (BP) may partially account for this variability.

Atrophy rate is a biomarker of AD progression, and an important outcome measure in clinical trials.^9^ It is necessary to understand how BP associates with atrophy rates to enable effective planning of clinical trials in which they are used as outcome measures.

Targeting BP in the same way across the cognitive spectrum may not be appropriate.^10^ Low BP is associated with poorer cognition in the elderly^11^ and is common in AD patients.^11^, ^12^ Falling BP is also associated with worsening cognition in the hypertensive elderly.^13^

Here, we aimed to understand relationships among late‐life BP, cognition, and brain volume in 700 participants from the Alzheimer's Disease Neuroimaging Initiative (ADNI). We also investigated BP and cognition in a group of 8240 participants from a complementary dataset: National Alzheimer's Coordinating Center (NACC). We hypothesized that BP would be associated with brain volume change; changes in BP would be associated with changes in brain volume. Similarly, we hypothesized that baseline BP would be associated with baseline neuropsychology and change in neuropsychology. We also considered that BP changes would be associated with changes in neuropsychology.

## 2 METHODS

### 2.1 Participants

#### 2.1.1 ADNI

We obtained data from the ADNI database (adni.loni.usc.edu). Data are freely available for qualified researchers. Participants engaged in baseline clinical, neuropsychometric, and magnetic resonance imaging (MRI) assessments and periodical assessments thereafter. Written informed consent was obtained from each participant and approved by the Institutional Review Board at each of the >50 participating centers. ADNI is a multicenter longitudinal private‐publicly funded study launched in 2003 investigating healthy controls, mild cognitive impairment (MCI), and AD participants. Based in America, ADNI is headed by Michael W. Weiner. The principle goal of ADNI has been to test whether use of serial MRI, positron emission tomography (PET), biomarkers, and clinical and neuropsychological data could measure the progression of MCI and early AD. For up‐to‐date information see www.adni-info.org. All participants were from the first phase of the study (ADNI1). All MCI and AD patients had an amnestic impairment. AD patients met criteria for probable AD according to the NINCDS‐ADRDA criteria.^14^ For in‐depth eligibility criteria, please see http://adni.loni.usc.edu/wp-content/uploads/2010/09/ADNI_GeneralProceduresManual.pdf and section 1.1 in the supporting information. In brief, all ADNI subjects had to be between 55 and 90 years at enrolment, be English or Spanish speaking, and have a partner able to provide an independent evaluation of functioning. Individuals taking specific psychoactive medications were excluded. We included patients enrolled in ADNI1 from September 2005 to 2010. The study schedule consisted of visits at baseline, 6 months, 12 months, 24 months, and 36 months. There were alterations to this schedule for disease groups; MCIs had an additional 18‐month visit and AD participants had their final visit at 24 months.

**RESEARCH IN CONTEXT**

1. Systematic review: We reviewed the literature using PubMed. There has been substantial recent interest in the relationships of blood pressure, cognition, and atrophy. The relevant publications have been cited in the manuscript.
2. Interpretation: Our findings demonstrate that potentially modifiable variables are associated with volume change in the brain in clinical Alzheimer's disease (AD). Our results also show there is a need to monitor blood pressure in late life including in individuals with cognitive impairment.
3. Future directions: Further research is required to (a) understand the mechanisms of late‐life hypertension and hypotension in neurodegeneration and cognitive impairment; (b) develop appropriate blood pressure levels for those in old age, and for those elderly with cognitive impairment; and (c) investigate whether stratification of clinical trials for putative AD treatments by blood pressure measurements is useful.

#### 2.1.2 NACC

We included participants from the NACC data set (http://www.alz.washington.edu/), from the March 2014 data freeze. Data are freely available for the research community. NACC has developed and maintains a database of standardized clinical research visits, collected from 39 past and present NIA‐funded Alzheimer's Disease Centers (ADCs) in the United States. The recruitment of participants and data collection in NACC has been described previously.^15^, ^16^ Institutional review boards at each ADC approved the study. Written and informed consent was given by all NACC participants.

We included patients seen at 34 ADCs from its inception in 2005 to February 2014. All participants visited for research purposes and were not enrolled in ADNI1 (Figure S1 in supporting information). Imaging data were unavailable for the majority of subjects; therefore, for NACC we investigated BP and cognition relationships only. Criteria on MCI diagnosis for ADCs was developed using consensus guidelines set by an expert panel.^17^ MCI patients were included if they had memory problems. AD patients were demented with a diagnosis of probable or possible AD at first visit.^14^ We excluded individuals with secondary causes of dementia (see section 1.1 in supporting information).

### 2.2 BP readings

Seated systolic blood pressure (SBP) and diastolic blood pressure (DBP) readings were measured at all visits for ADNI and at approximately annual visits for NACC (see section 1.2 in supporting information). Pulse pressure (PP) was calculated as SBP minus DBP.

### 2.3 Image analysis

The ADNI MRI protocol is detailed elsewhere.^18^ After the acquisition, quality control was completed and ADNI image pre‐processing was then applied, including gradient warping,^19^ B1 non‐uniformity,^20^ and intensity non‐uniformity correction.^21^ Imaging data consisted of all available ADNI1 time points from baseline to 36 months (0‐, 6‐, 12‐, 18‐, 24‐, and 36‐month scans), with a T1‐weighted volumetric scan acquired on a 1.5T scanner of sufficient quality. Internal visual quality control was performed, excluding images with severe blurring of tissue boundaries due to motion artefacts. Whole‐brain and hippocampal volumes were estimated automatically from the 1.5T volumetric T1‐weighted images using BMAPS^22^ and HMAPS, respectively.^23^ The boundary shift integral (BSI) was used to estimate change directly from scan pairs after segmentation,^24^ the outcome representing change in volume of whole‐brain or hippocampus (mL) during the scan interval. Total intracranial volume (TIV), a proxy for head size, was also calculated.^25^

### 2.4 Cognition

We investigated three longitudinal cognition outcomes: Mini‐Mental State Examination (MMSE) for global impairment, Logical Memory Immediate Story Recall (LM) for memory, WAIS‐R Digit Symbol (DSST) for processing speed. Examinations were administered at each visit, apart from LM in ADNI, which was administered annually.

### 2.5 Statistical models

#### 2.5.1 Demographic information

Demographic differences between groups for continuous outcomes were tested using analysis of variance; Fisher's exact test was used for categorical outcomes. TIV was used as a covariate for brain and hippocampal outcomes.

#### 2.5.2 Joint modeling of outcomes

We jointly modeled change in BP (using SBP, DBP, and PP in separate models), change in cognition, and change in brain/hippocampal volume. There was no “predictor” as with traditional regression models; to observe concurrent changes in BP, cognition, and atrophy, each of the three variables were jointly modeled as outcomes. Multivariate mixed linear‐regression models were fitted with outcomes of BP (SBP, DBP, PP), atrophy (whole‐brain, hippocampal change), and cognition (MMSE, LM, DSST). A random intercept (BP, atrophy rate, and cognition) and random slope were included for the participant level with unstructured covariance to allow for correlations among all random effects, apart from the random intercept for atrophy rate. The atrophy rate random intercept was included to account for the random measurement error at the first scan, which would be shared between all atrophy measures. Likelihood ratio tests were used to test random effects correlations between outcomes. The following correlations were examined: (1) baseline BP, change in brain volume; (2) change in BP, change in brain volume; (3) baseline BP, baseline neuropsychology; (4) baseline BP, change in neuropsychology; (5) change in BP, change in neuropsychology. Models were run separately in each diagnostic group.

No fixed effects intercept was included in the model for change in brain volume due to the assumption that the estimated atrophy rate over a scan interval of zero is zero. As no intercept, or measure of baseline volume change, is estimated using BSI (volume change) data, we separately modeled baseline brain and hippocampal volume in a regression model (see below). We used the multilevel modeling software MLWin version 2.36, February 2016 release.^26^ For BP and cognitive models, age and sex were added as covariates to adjust for their associations with the baseline value of these outcomes, and for all outcomes these covariates were interacted with time, to adjust for their associations with changes in outcome. Apolipoprotein E (*APOE*) ε4 status (binary covariate indicating presence or absence of an ε4 allele) was included in the relevant ADNI models to adjust for associations with baseline BP, baseline MMSE, MMSE change, and brain volume change.

Fixed effects data are tabulated in supporting information. Due to lack of any detectable change over time in SBP and DBP for ADNI AD patients the random effect slope for change in blood pressure was removed to allow the model to converge. For PP in the ADNI AD group the random effect slope was permitted, but the random effect correlation between baseline PP and change in PP was omitted.

For NACC, models were also fitted with adjustment for antihypertensive medication use and also for *APOE*ε4 (binary covariate indicating presence or absence of an ε4 allele), see section 1.3 in supporting information.

In ADNI, to investigate correlations between baseline BP and baseline whole‐brain/hippocampal volumes, regression models were fitted, with separate outcomes of whole‐brain/hippocampal volume and baseline BP (either SBP, DBP, or PP) as the predictor. Age, *APOE* ε4 status, TIV, and sex were included as covariates.

#### 2.5.3 Analysis by hypertensive status in AD

AD patients were categorized by hypertensive status based upon initial BP and antihypertensive usage. BP cut‐offs for hypertension were defined as = >140 mmHg for SBP and/or = >90 mmHg for DBP.^27^ Hypotension was defined as SBP = < 90 mmHg and/or = <60 mmHg for DBP, as defined by the United States Institutes of Health (https://www.nhlbi.nih.gov/health-topics/hypotension). Demographics for groups and fixed effects resulting from the models are tabulated in Table S7 in supporting information.

For information on figures and tabulation see section 1.4 in supporting information. For reference we ran all graphically presented effects without adjustment for covariates.

## 3 RESULTS

### 3.1 Demographic information

After quality control, 700 ADNI participants were included in this study (see Figure S1). MCI and AD patients had lower brain and hippocampal volumes, greater cognitive impairment, and were more likely to be an *APOE* ε4 carrier than controls (Table 1).

**Table 1:** Subject demographics and basic imaging information for the Alzheimer's disease Neuroimaging Initiative (ADNI) and National Alzheimer's Co‐ordinating Center (NACC) cohorts

	ADNI				NACC			
	Controls	Mild cognitive impairment	Alzheimer's disease	Group difference (*P* value)	Controls	Mild cognitive impairment	Alzheimer's disease	Group difference (*P* value)
N	200	346	154		1098	2297	4845	
Follow‐up length (years)	2.6 (0.8)	2.3 (0.8)	1.7 (0.6)	<0.01	4.5 (1.8)	2.4 (2.0)	1.4 (1.7)	<0.01
Number of time points (min., max.)	3.2 (1, 4)	3.6 (1, 5)	2.3 (1, 3)	<0.01	4.9 (1, 7)	3.0 (1, 7)	2.3 (1, 7)	<0.01
Age at baseline (years)	76.0 (5.2)	75.0 (7.2)	75.0 (7.7)	0.22	78.1 (8.7)	75.3 (9.1)	75.5 (10.0)	<0.01
Percentage male	53	63	54	0.02	38	50	47	<0.01
Percentage *APOE*ε4^^^	26	55	69	<0.01	36	45	59	<0.01
Years of education	16.1 (2.8)	15.7 (3.0)	14.8 (3.0)	<0.01	15.8 (7.4)	15.4 (5.4)	14.9 (8.7)	<0.01
Total brain vol. (mL)	1068.0 (103.1)	1061.9 (114.6)	1022 .0 (115.1)	<0.01#	n/a			
Total hippocampal vol. (mL)	5.2 (0.7)	4.5 (0.8)	3.9 (0.9)	<0.01#	n/a			
Baseline SBP* (mmHg)	133.9 (16.3)	134.4 (17.9)	137.5 (17.2)	0.1	135.2 (18.2)	136.0 (18.9)	134.9 (19.2)	0.09
Baseline DBP* (mmHg)	74.4 (10.3)	74.5 (9.5)	74.4 (9.5)	0.9	73.5 (10.2)	74.8 (10.4)	74.5 (10.9)	<0.01
Baseline pulse pressure* (mmHg)	59.4 (15.2)	59.9 (15.8)	63.1 (16.5)	0.07	61.7 (16.5)	61.2 (16.6)	60.4 (17.0)	0.03
Baseline MMSE, /30	29.1 (1.0)	27.0 (1.8)	23.4 (1.9)	<0.01	28.5 (1.6)	26.9 (2.5)	19.7 (6.8)	<0.01
Baseline LM, /25	13.9 (3.4)	7.1 (3.1)	3.9 (2.8)	<0.01	12.1 (3.9)	8.4 (4.0)	4.0 (3.6)	<0.01
Baseline DSST**, /93	46.0 (10.2)	37.0 (11.2)	27.5 (12.4)	<0.01	41.0 (11.8)	37.1 (12.0)	24.8 (14.1)	<0.01
Percentage hypercholesteremic***	25	30	36	0.09	45	52	47	0.04^
Percentage diabetic ▲	6	7	6	0.9	10	13	12	<0.001^
Percentage smoker (past or current) ▲▲	40	42	40	0.9	42	46	43	0.03
Percentage on antihypertensive medication at baseline ▲▲▲	n/a				62%	60%	54%	<0.01

Values are mean (standard deviation) unless reported. Systolic blood pressure (SBP) and diastolic blood pressure (DBP), MMSE (Mini‐Mental State Examination), LM (logical immediate story recall) and DSST (WAIS‐R Digit Symbol) are investigated. # Adjusted for TIV. ^Data missing in 199 NACC control subjects, 873 NACC mild cognitive impairment patients and 1861 NACC Alzheimer's disease patients. ^*^data missing in 91 NACC subjects. ^**^data missing in 63 ADNI subjects, ^***^data missing in 90 NACC subjects, ▲ data missing in 35 NACC subjects, ▲▲ data missing in 120 NACC subjects, ▲▲▲ data missing in 97 NACC subjects. The proportion of NACC individuals with hypertensive baseline BP readings, who were not on antihypertensive medication was calculated as a percentage. ^ figures and p value exclude subjects with remote or inactive hypercholesterolemia (remote/inactive hypercholesterolemia 3% for controls; 3% MCI; 4% AD) or remote/inactive diabetes (0.4% control, 1% MCI, 1% AD) for NACC participants.

A total of 8240 NACC participants were suitable for the present study. Controls were older and were more likely to be female, compared with MCI and AD groups.

### 3.2 Control results

From both ADNI and NACC, controls on average had no significant change in BP over time (see Table S1 in supporting information). ADNI controls had significant rates of brain and hippocampal volume loss, but did not decline in cognition over time. The larger group of NACC controls had, on average, a decline in MMSE and LM.

#### 3.2.1 BP and atrophy

**FIGURE 1:**
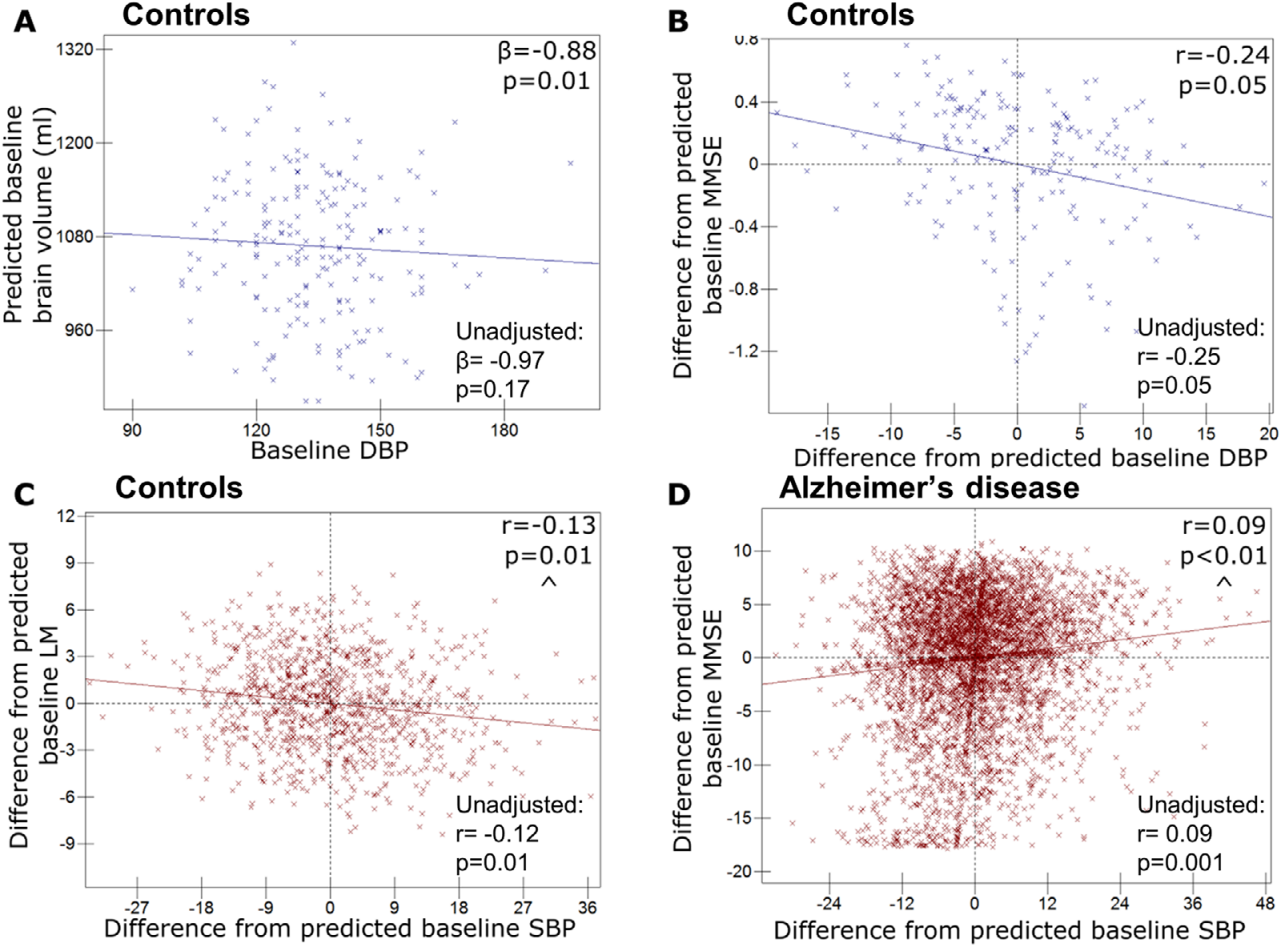
Relationships between baseline blood pressure (BP) and baseline psychology or hippocampal volume. Graphs to demonstrate relationships between baseline blood pressure and baseline psychology or hippocampal volume (Alzheimer's Disease Neuroimaging Initiative [ADNI] denoted by blue crosses and NACC [National Alzheimer's Co‐ordinating Center] by red). Predicted values from linear regression of baseline diastolic BP (DBP) and baseline whole‐brain volume in ADNI control participants (a); All others, graphs of participant level residuals, demonstrating random effect correlations between: (B) baseline DBP and baseline Mini‐Mental State Examination (MMSE) in ADNI controls; (C) baseline systolic BP (SBP) and baseline Logical Memory Immediate Story recall (LM) in NACC controls; (D) baseline SBP and baseline MMSE in NACC Alzheimer's disease patients. All ADNI relationships are corrected for *APOE* genotype, age, sex, and total intracranial volume. All NACC relationships adjusted for age and sex. Results for models without covariate adjustment are also shown. ^ Similar relationship exists for pulse pressure.

ADNI control participants with higher baseline DBP had lower baseline brain volumes, see Figure 1a, Table S2 in supporting information, corresponding to a ‐0.88 mL decrease for a 1 mmHg increase in diastolic BP, from the average brain volume of 1132 mL (estimate, [95%CI] (*P* value); (–0.88 [–1.59, –0.17] (0.01)). However, there were no significant correlations between baseline, or change in, blood pressure and brain/hippocampal volume change over time (Table S3 in supporting information).

#### 3.2.2 BP and neuropsychology

In both cohorts, higher BP tended to be associated with worse baseline neuropsychology scores in controls, although this was not found across all measures. In ADNI, controls with higher baseline DBP had a worse baseline MMSE score (correlation coefficient, *P* value) (–0.24, 0.05) (see Figure 1B, Table S4 in supporting information). In NACC, controls with higher SBP had lower baseline LM (–0.13, 0.01) (see Table 2, Figure 1C). NACC controls with higher PP also had lower DSST scores (–0.12, 0.01). No correlations were found between changes in BP and changes in neuropsychology.

**Table 2:** Table showing correlations between blood pressure (BP) (systolic blood pressure [SBP], diastolic blood pressure [DBP], and pulse pressure [PP]) and neuropsychology in the NACC dataset

NACC		Controls			Mild cognitive impairment			Alzheimer's disease		
Correlation of interest	BP variable	Correlation with neuropsychology								
		MMSE	LM	DSST	MMSE	LM	DSST	MMSE	LM	DSST
Baseline BP, Baseline cognition	SBP	−0.09 (0.17)	−0.13 (0.01)	−0.07 (0.12)	−0.11 (0.01)	−0.09 (0.01)	−0.04 (0.2)	0.09 (0.0003)	0.01 (0.65)	0.00 (1)
	DBP	0.00 (0.96)	−0.05 (0.29)	0.05 (0.3)	−0.05 (0.24)	−0.04 (0.29)	−0.01 (0.68)	0.02 (0.31)	−0.03 (0.24)	−0.04 (0.18)
	PP	−0.12 (0.09)	−0.12 (0.02)	−0.12 (0.01)	−0.10 (0.02)	−0.08 (0.03)	−0.04 (0.2)	0.09 (0.0005)	0.04 (0.2)	0.02 (0.45)
Baseline BP, Change in cognition	SBP	−0.03 (0.59)	−0.02 (0.73)	−0.02 (0.72)	−0.08 (0.08)	−0.04 (0.56)	−0.12 (0.02)	0.00 (0.93)	0.02 (0.74)	−0.02 (0.72)
	DBP	−0.04 (0.42)	−0.06 (0.31)	−0.08 (0.24)	0.06 (0.19)	0.14 (0.02)	0.02 (0.76)	−0.03 (0.4)	0.05 (0.43)	−0.01 (0.85)
	PP	0.00 (0.97)	0.02 (0.77)	0.03 (0.7)	−0.14 (0.003)	−0.14 (0.02)	−0.16 (0.003)	0.02 (0.67)	−0.01 (0.93)	0.00 (1)
Change in BP, Change in cognition	SBP	0.07 (0.25)	0.00 (0.97)	0.01 (0.95)	0.28 (0.07)	0.20 (0.06)	0.43 (0.0001)	0.19 (0.003)	0.05 (0.82)	0.09 (0.5)
	DBP	0.05 (0.62)	0.03 (0.8)	−0.01 (0.91)	0.03 (0.73)	−0.06 (0.62)	0.17 (0.1)	0.14 (0.18)	−0.05 (0.27)	−0.05 (0.66)
	PP	0.05 (0.1)	−0.03 (0.83)	0.02 (0.89)	0.32 (0.01)	0.28 (0.01)	0.40 (0.00003)	0.14 (0.001)	0.11 (0.3)	0.11 (0.44)

Random effects correlations (r and [*P* values]) are shown. BP and neuropsychology were jointly modeled as outcomes for each type of BP measurement and for each neuropsychology test (MMSE [Mini‐Mental State Examination], LM [logical immediate story recall], and DSST [WAIS‐R Digit Symbol]). Analyses are adjusted for sex and age.

### 3.3 MCI results

In both ADNI and NACC, MCI participants had falling DBP over time (Table S1). ADNI MCI participants declined in MMSE and DSST over time, but not LM. NACC MCI participants showed significant rates of decline in all neuropsychology measures.

#### 3.3.1 BP and atrophy

**FIGURE 2:**
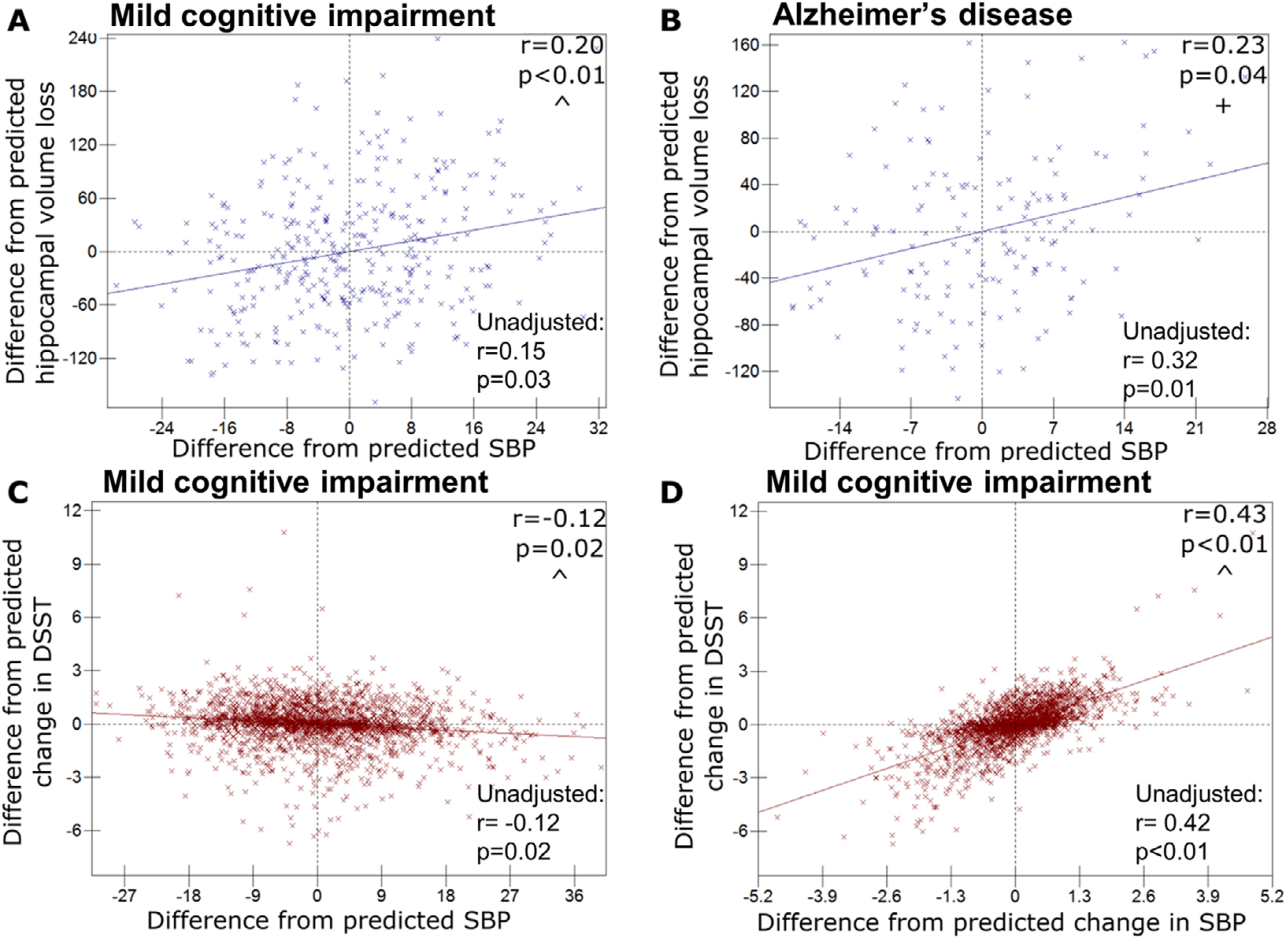
Relationships between blood pressure (BP) and cognitive or hippocampal change. Graphs of participant level residuals, demonstrating random effect correlations between baseline or change in BP and cognitive or hippocampal change (Alzheimer's Disease Neuroimaging Initiative [ADNI] denoted by blue crosses and NACC [National Alzheimer's Co‐ordinating Center] by red). Baseline systolic BP (SBP) and hippocampal volume loss in ADNI mild cognitive impairment (mild cognitive impairment) patients (A), and Alzheimer's disease patients (B); (C–D) NACC mild cognitive impairment patients; (C) baseline SBP and change in Digit Symbol (DSST); (D) SBP change and DSST change. All ADNI relationships are corrected for *APOE* genotype, age, sex, and total intracranial volume. All NACC relationships adjusted for age and sex. Results for models without covariate adjustment are also shown. ^Similar relationship exists for pulse pressure; + similar relationship exists for diastolic blood pressure

For ADNI MCI patients, higher baseline SBP was associated with higher hippocampal atrophy rates (correlation coefficient, *P* value) (0.20, 0.005) (see Table 3, Figure 2A) and those with higher PP also had greater hippocampal atrophy rates (0.21, 0.004). No significant correlations were seen between baseline BP, or changes in BP, and whole brain atrophy rates.

**Table 3:** Systolic blood pressure (SBP), diastolic blood pressure (DBP), and pulse pressure (PP) and hippocampal volume change are correlated in Alzheimer's Disease Neuroimaging Initiative (ADNI1)

ADNI BP and hippocampal atrophy rate correlation	Controls	Mild cognitive impairment	Alzheimer's disease
Baseline SBP	0.01 (0.96)	0.20 (0.005)	0.23 (0.04)
Baseline DBP	−0.12 (0.24)	0.04 (0.57)	0.22 (0.05)
Baseline PP	0.08 (0.41)	0.21 (0.004)	0.19 (0.14)
Change in SBP	−0.10 (0.48)	−0.02 (0.83)	Inestimable
Change in DBP	0.32 (0.17)	−0.06 (0.67)	Inestimable
Change in PP	−0.25 (0.10)	0.00 (0.96)	−0.46 (0.19)

Random effects correlations (r and [*P* values]) are shown. Analyses are adjusted for sex, age, *APOE* ε4 genotype, and total intracranial volume.

#### 3.3.2 BP and neuropsychology

In NACC, higher baseline SBP showed significant correlations with lower baseline MMSE (−0.11, 0.01) (see Table 2, Figure S2a in supporting information), and LM (−0.09, 0.01), and faster decline in DSST (−0.12, 0.02) (Figure 2C). Low DBP was related to a decline in LM (0.14, 0.02) (Figure S2b in supporting information). Higher baseline PP had significant associations with lower baseline MMSE (−0.10, 0.02) and LM (−0.08, 0.03), and decline in MMSE (−0.14, 0.003), DSST (−0.16, 0.003), and LM (−0.14, 0.02) (Figure S2c).

Falling SBP was associated with declining DSST (0.43, 0.0001) (Figure 2D), and a similar effect was seen with falling PP and declining DSST (0.40, 0.00003), MMSE (0.32, 0.01), and LM (0.28, 0.01).

No correlations were found between BP and neuropsychology in ADNI (see Table S4).

### 3.4 AD results

The ADNI AD group did not show any significant change over time in BP, but falling SBP, DBP, and PP were all detected in the NACC AD group, see Table 1.

#### 3.4.1 BP and atrophy

Higher baseline SBP was associated with greater subsequent hippocampal atrophy (0.23, 0.04) (see Table 3, Figure 2B); this relationship was mirrored by DBP (0.22, 0.05) (see Table 3, Figure S2d). It was not possible to examine associations between atrophy rates and changes for SBP or DBP due to minimal between‐participant variability in change over time in these measures. No association was found between change in PP and either hippocampal or whole brain atrophy rate.

#### 3.4.2 BP and neuropsychology

In ADNI, no significant correlations with neuropsychology were observed. However, in NACC, higher SBP pressure and higher PP were associated with higher baseline MMSE (0.09, 0.0003 [Figure 1D) and 0.09, 0.0005 respectively). Falling SBP and declining PP were associated with faster declines in MMSE (0.19, 0.003 [Figure S2e] and 0.14, 0.001, respectively).

#### 3.4.3 Analysis of AD patients by hypertensive group and medication

We found low baseline BP predicted low baseline MMSE in the medicated hypotensives (see Table 4) ([correlation coefficient, *P* value] 0.37, 0.01), and the non‐medicated, normotensives (0.21, 0.006). A relationship between low baseline BP and subsequent MMSE decline was found in hypotensive groups (non‐medicated [0.59, 0.04] and medicated [0.43, 0.03]). An opposing relationship was found in the hypertensive groups, in which high baseline BP predicted MMSE decline, although only significantly in the non‐medicated group despite equal effect sizes (non‐medicated [–0.26, 0.02], medicated [–0.26, 0.13]). Last, both hypertensive groups showed simultaneously falling BP and falling MMSE (non‐medicated [0.58, 0.001], medicated [0.82, 0.03]). There were no relationships between BP and cognition in the medicated normotensives, despite being the largest group.

**Table 4:** Results for Alzheimer's disease patients split by baseline hypertensive status, random effects correlations resulting from joint models of systolic blood pressure (SBP) and Mini‐Mental State Examination (MMSE) change in AD patients by hypertensive status

	Hypotensive		Normotensive		Hypertensive	
NACC	Non‐medicated	Medicated	Non‐medicated	Medicated	Non‐medicated	Medicated
1. Non‐medicated hypotensive	2. Medicated hypotensive	3. Non‐medicated normotensive	4. Medicated normotensive	5. Non‐medicated hypertensive	6. Medicated hypertensive	
N	103	156	1168	2084	928	374
Baseline BP, Baseline MMSE correlation	0.10 (0.56)	0.37 (0.01)	0.21 (0.006)	0.02 (0.56)	0.09 (0.29)	0.02 (0.88)
Baseline BP, Change in MMSE correlation	0.59 (0.04)	0.43 (0.03)	0.11 (0.33)	−0.01 (0.83)	−0.26 (0.02)	−0.26 (0.13)
Change in BP, Change in MMSE correlation	−0.55 (0.11)	Inestimable	−0.02 (0.92)	0.17 (0.33)	0.58 (0.001)	0.82 (0.03)

Correlation (r and [*P* values]) are shown for correlations between SBP and MMSE. Analyses are adjusted for sex and age.

### 3.5 Adjustment for *APOE* genotype and antihypertensive use

With adjustment for *APOE* ε4 genotype in NACC the overall relationships between BP and cognition were not materially changed (Table S5 in supporting information). With adjustment for antihypertensive usage in NACC (Table S6 in supporting information) all relationships remained unchanged, apart from the correlation between declining SBP and PP and falling MMSE in AD, which was no longer significant.

Graphically represented results were also tested without adjustment for covariates and the majority of findings were materially unchanged (see Figures 1 and 2 and Figure S2). The relationship between DBP and atrophy rate in AD participants changed to trend level without covariate adjustment (Figure S2). Without covariate adjustment the relationship between DBP and brain volume in controls was no longer significant (Figure 1), and the relationship between change in MMSE and SBP in AD was no longer significant (Figure S2); however, for both results the effect size and direction remained similar.

## 4 DISCUSSION

The central finding of this study is that higher baseline BP is associated with greater hippocampal atrophy rates in MCI and AD. This is the first demonstration, to our knowledge, of a potentially modifiable risk factor which is associated with hippocampal atrophy rate in clinical AD. In AD, higher baseline BP appeared detrimental to future cognition in hypertensive patients, whereas low BP was predictive of cognitive decline in hypotensives. In hypertensive ADs, falling BP and MMSE occurred simultaneously.

The novel observation that higher baseline BP predicts greater hippocampal atrophy rate in MCI and AD has potential clinical implications. Few factors have been found which determine atrophy rate in AD, and those that have are not currently amenable to intervention: *APOE* ε4 genotype, sex, baseline atrophy level, and AD pathological load.^28^, ^29^, ^30^, ^31^, ^32^ Clinical trials targeting amyloid pathology have, to date, not shown a beneficial impact on atrophy rates or cognition in symptomatic individuals.^33^ Licensed antihypertensives are available, meaning BP control may be a route to attenuating decline. However, because falls of BP in hypertensive AD groups were associated with worsening cognitive scores, more research is required to examine whether active lowering of BP is appropriate.

BP in late‐life has been inconsistently related to cognition and cognitive decline.^10^ High BP has been identified as a risk factor for lower MMSE score^10^ and smaller whole brain volume.^34^ Conversely, low BP has been found to predict greater longitudinal atrophy in cognitively normal individuals with manifest arterial disease.^5^ We did not find high BP predicted atrophy rate in controls, similarly to others^35^, ^36^, ^37^ although some have detected relationships.^6^, ^7^ The duration of hypertension may explain why dementia is most consistently associated with mid‐, rather than late‐life BP.

SBP appeared to have the strongest influence on brain volumes and cognition across the cohorts. Many relationships between SBP and cognition in NACC were mirrored by similarly strong effects in PP. PP is considered an indirect measure of arterial stiffness, and has also been shown to negatively influence brain volumes and cognition.^38^

We found the impact of BP on cognition was dependent on disease stage; low BP correlated with poor cognition in AD, whereas in controls and MCI high BP predicted lower cognition. Falling BP tracked with declining MMSE in both MCI and AD. Low BP has previously been found to relate to poorer cognition^12^ and is frequently observed in old AD patients.^11^, ^12^ At the prodromal AD stage, high BP may be harmful, potentially lowering the threshold at which symptoms appear (perhaps through cerebrovascular disease); falling and subsequently low BP in symptomatic AD may then reflect disease stage. Studies investigating long‐term BP trajectories have found that hypertension, followed by declining BP, and subsequent hypotension is associated with lower cross‐sectional brain volumes^39^, ^40^ and is common in those who develop dementia.^41^ One study showed that increases in BP from ages 36 to 43 years were associated with lower brain volumes at 70 years indicating that monitoring of BP needs to happen from early adulthood.^42^ Low BP is likely to impair cerebral perfusion, which may be worse in those with histories of hypertension, due to arterial stiffening and impaired cerebro‐autoregulation.^43^ Elderly individuals may also require a higher BP to maintain adequate perfusion.^12^ Conversely, low BP could also be a product of AD‐related neurodegeneration in BP‐regulating brain areas.^44^

When the NACC AD group was categorized dependent on baseline hypertensive status, a pattern emerged which supports careful BP monitoring. In non‐medicated hypertensives, high baseline SBP was detrimental for future cognition, whereas in hypotensives and non‐medicated normotensives, low SBP was predictive of cognitive decline. Interestingly, those with successfully lowered BP were the only group without relationships between BP and cognition, despite being the largest group. Notably in this group, baseline SBP was more than 10mmHg higher than the non‐medicated normotensives; a higher BP may be necessary to maintain adequate brain perfusion in old age.^12^

Using data from two cohorts, our study was large, with a combined total of 8940 participants. Our models investigated longitudinal data from three outcomes, which enabled detection of joint patterns at an individual level. We also investigated relationships across the cognitive spectrum. While we ran a relatively large number of models, we opted not to correct for multiple comparisons, as our tests were hypothesis driven. Follow‐up was relatively short in both cohorts. It may be that decades of follow‐up are needed to fully understand the relationships among BP, brain volumes, and cognition. Both cohorts have limitations in generalizability because subjects enrolled in studies tend to be younger and fitter than those in the community.^45^, ^46^, ^47^ Specifically to ADNI, subjects must score ≤4 on the Hachinski scale,^48^ meaning their vascular health may be better than the general population. Different inclusion criteria were used in each cohort and these may influence the results seen; for example, it may be that the likely differing amounts of cerebrovascular disease between cohorts influenced findings. Possible differences in BP measurement protocols between the cohorts may have further influenced results. We chose three neuropsychology tests relevant for AD; however, further tests are available and could be investigated. Further, we chose to investigate SBP, DBP, and PP as they are more immediately applicable for health‐care professionals; other BP measures would be interesting to investigate.

Late‐life high BP is the only known modifiable predictor of atrophy rate (change in volume) in clinically defined AD. More work is required to understand whether lowering of BP can attenuate progression in hypertensive cognitively impaired individuals. For clinical trials targeting AD pathology, increased power to detect a treatment effect may be achieved by stratifying or adjusting for BP. Last, in AD, both hypertension and hypotension may accelerate disease course, and therefore BP should be carefully monitored.

## CONFLICTS OF INTEREST

There are no reported conflicts apart from a note that Christopher Lane is now employed by Eisai Europe Ltd.

## FUNDING INFORMATION

The Dementia Research Centre is supported by Alzheimer's Research UK, Brain Research Trust, and The Wolfson Foundation. This work was supported by the NIHR Queen Square Dementia Biomedical Research Unit and the National Institute for Health Research Biomedical Research Centre (BRC). CF and JB are supported by Alzheimer's Research UK; Alzheimer's Research UK Senior Fellowship (ARUK‐SRF2016A‐2). Christopher Lane was funded by an MRC DPUK grant.

Data collection and sharing for this project was funded by the (ADNI) (National Institutes of Health Grant U01 AG024904) and DOD ADNI (Department of Defense award number W81XWH‐12‐2‐0012). ADNI is funded by the National Institute on Aging, the National Institute of Biomedical Imaging and Bioengineering, and through generous contributions from the following: AbbVie, Alzheimer's Association; Alzheimer's Drug Discovery Foundation; Araclon Biotech; BioClinica, Inc.; Biogen; Bristol‐Myers Squibb Company; CereSpir, Inc.; Cogstate; Eisai Inc.; Elan Pharmaceuticals, Inc.; Eli Lilly and Company; EuroImmun; F. Hoffmann‐La Roche Ltd and its affiliated company Genentech, Inc.; Fujirebio; GE Healthcare; IXICO Ltd.; Janssen Alzheimer Immunotherapy Research & Development, LLC.; Johnson & Johnson Pharmaceutical Research & Development LLC.; Lumosity; Lundbeck; Merck & Co., Inc.; Meso Scale Diagnostics, LLC.; NeuroRx Research; Neurotrack Technologies; Novartis Pharmaceuticals Corporation; Pfizer Inc.; Piramal Imaging; Servier; Takeda Pharmaceutical Company; and Transition Therapeutics. The Canadian Institutes of Health Research is providing funds to support ADNI clinical sites in Canada. Private sector contributions are facilitated by the Foundation for the National Institutes of Health (www.fnih.org). The grantee organization is the Northern California Institute for Research and Education, and the study is coordinated by the Alzheimer's Therapeutic Research Institute at the University of Southern California. ADNI data are disseminated by the Laboratory for Neuro Imaging at the University of Southern California.

The NACC database is funded by NIA/NIH Grant U01 AG016976. NACC data are contributed by the NIA funded ADCs: P30 AG019610 (PI Eric Reiman, MD), P30 AG013846 (PI Neil Kowall, MD), P50 AG008702 (PI Scott Small, MD), P50 AG025688 (PI Allan Levey, MD, PhD), P50 AG047266 (PI Todd Golde, MD, PhD), P30 AG010133 (PI Andrew Saykin, PsyD), P50 AG005146 (PI Marilyn Albert, PhD), P50 AG005134 (PI Bradley Hyman, MD, PhD), P50 AG016574 (PI Ronald Petersen, MD, PhD), P50 AG005138 (PI Mary Sano, PhD), P30 AG008051 (PI Thomas Wisniewski, MD), P30 AG013854 (PI M. Marsel Mesulam, MD), P30 AG008017 (PI Jeffrey Kaye, MD), P30 AG010161 (PI David Bennett, MD), P50 AG047366 (PI Victor Henderson, MD, MS), P30 AG010129 (PI Charles DeCarli, MD), P50 AG016573 (PI Frank LaFerla, PhD), P50 AG005131 (PI James Brewer, MD, PhD), P50 AG023501 (PI Bruce Miller, MD), P30 AG035982 (PI Russell Swerdlow, MD), P30 AG028383 (PI Linda Van Eldik, PhD), P30 AG053760 (PI Henry Paulson, MD, PhD), P30 AG010124 (PI John Trojanowski, MD, PhD), P50 AG005133 (PI Oscar Lopez, MD), P50 AG005142 (PI Helena Chui, MD), P30 AG012300 (PI Roger Rosenberg, MD), P30 AG049638 (PI Suzanne Craft, PhD), P50 AG005136 (PI Thomas Grabowski, MD), P50 AG033514 (PI Sanjay Asthana, MD, FRCP), P50 AG005681 (PI John Morris, MD), P50 AG047270 (PI Stephen Strittmatter, MD, PhD).

## Supporting information

Supporting Information.
