# Supplementary material for: High blood pressure predicts hippocampal atrophy rate in cognitively impaired elders
Source: Alzheimers Dement (Amst). 2020 May 17;12(1):e12035. doi: 10.1002/dad2.12035 (PMC7308793; doi:10.1002/dad2.12035)
Supplement: Supplementary file 1 — Supporting Information. [file DAD2-12-e12035-s001.docx]

**SUPPLEMENTARY MATERIAL**

High blood pressure predicts hippocampal atrophy rate in cognitively impaired elders

**Supplementary Methods**

Section 1.1 General inclusion criteria

Alzheimer’s Disease Neuroimaging Initiative (ADNI, taken from the procedures manual):

For all subjects they must:

* have a Hachinski score less than or equal to 4

* be between 55-90 years old

* be stable on specific allowed medications for at least 4 weeks

* have a Geriatric Depression Scale score lower than 6

* have a study partner with at least 10 hr/wk contact and who accompanies the participant to visits

* have good visual and auditory acuity to perform neuropsychological tests

* be in good general health with no diseases precluding enrolment

* be sterile or at least two years past childbearing potential if female

* be willing and judged able to complete a 3-year study with imaging (2 years for individuals AD)

* have 6 grades of education or work history

* be fluent in English or Spanish

* be willing to have neuroimaging and without contraindications to MRI

* agree to having DNA taken for ApoE testing and banking

* agree to blood and urine being taken and assessed for biomarkers

* not be enrolled in other trials or studies

For specific groups:

Normal subjects: MMSE scores between 24-30 (inclusive), a CDR of 0, non-depressed, non MCI, and non-demented.

MCI subjects: MMSE scores between 24-30 (inclusive), a memory complaint verified by study partner, have objective memory loss measured by education adjusted scores on Wechsler Memory Scale Logical Memory II, a CDR of 0.5, absence of significant levels of impairment in other cognitive domains, essentially preserved activities of daily living, and an absence of dementia.

AD: MMSE scores between 20-26 (inclusive), a memory complaint verified by study partner, have objective memory loss measured by education adjusted scores on Wechsler Memory Scale Logical Memory II, CDR of 0.5 or 1.0, and meets NINCDS/ADRDA criteria for probable AD (McKhann et al, Neurology 1984; 34: 939-44).

National Alzheimer’s Co-ordinating Centre (NACC, taken from the NACC Uniform Data Set – Initial Visit Packet and Coding Guidebook)

Controls: subjects to have normal cognition (no MCI, dementia or other neurological condition resulting in cognitive impairment).

MCI: subjects had to have memory problems and no dementia. Their memory problems had to be abnormal for age with cognitive decline but essentially normal functional activities.

AD: subjects to be demented and have a diagnosis of possible or probable Alzheimer’s disease according to NINCDS/ADRDA criteria (McKhann et al, Neurology 1984; 34: 939-44). Subjects with AD were excluded if they were thought to also have: dementia with Lewy bodies, vascular dementia, alcohol-related dementia, dementia of undetermined aetiology, frontotemporal dementia, primary progressive aphasia, progressive supranuclear palsy, corticobasal degeneration, Huntington’s disease, prion disease, cognitive dysfunction from medications or medical illnesses, depression, other major psychiatric illness, Down’s syndrome, Parkinson’s disease, stroke, hydrocephalus, traumatic brain injury, central nervous system neoplasm or any other alternative specified condition that may cause the cognitive impairment.

Section 1.2 Blood pressure measurement

ADNI: The protocol states that investigators should see individuals subject at approximately the same time of day. No conversation should be had before or during the measurements. Measurements should be taken in the same, preferably dominant, arm ideally by the same person using the same calibrated device. When seated the forearm should be at the horizontal level of the fourth intercostal space at the sternum (at the level of the heart).

NACC: The forms state that the blood pressure is taken in the sitting position

Section 1.3 Antihypertensive adjustment

NACC models were adjusted for anithypertensive medication use (a binary longitudinal variable indicating use of an antihypertensive medication at each timepoint). The following BP lowering agents were considered: angiotensin converting enzyme inhibitor, angiotensin II receptor blocker, adrenergic agent, beta blocker, calcium channel blocking agent, diuretic, vasodilator. Notably, we did not take into account whether patients were prescribed these as antihypertensives, therefore many may be taking these medications for other reasons

Section 1.4 Tabulation and Graphing

Models were repeated substituting one outcome for another, all models and combinations came to 36 models for Alzheimer’s disease neuroimaging initiative (ADNI) and nine for NACC. Fixed effect data is tabulated in the supplementary, as it is not required for interpretation of the main outcomes. Correlations are our primary outcome and each correlation is between two variables. However, each model is constructed with three different outcomes (a neuropsychology outcome, a blood pressure (BP) value and a whole-brain/hippocampal volume change value). The results for correlation between two variables are very similar when the third variable is changed. For example the relationship between brain volume change and BP are highly similar when either LM, DSST or MMSE are used the third outcome; results are therefore tabulated with MMSE as the third variable. Likewise the correlations between each psychology test and BP are very similar whether hippocampal volume or whole-brain volume is the third variable, therefore for correlations between each neuropsychology test and BP, whole-brain volume is the third outcome. Baseline MMSE predicted from the fixed effect portion of the model (supplementary table 1, may vary from the baseline average MMSE in the demographic table (main paper table 1). Residuals from statistically significant random effects correlations (p<0.05) were plotted. One example of each of the main findings of the paper is graphically displayed in the paper, further examples of each relationship are illustrated in supplementary figure 2.


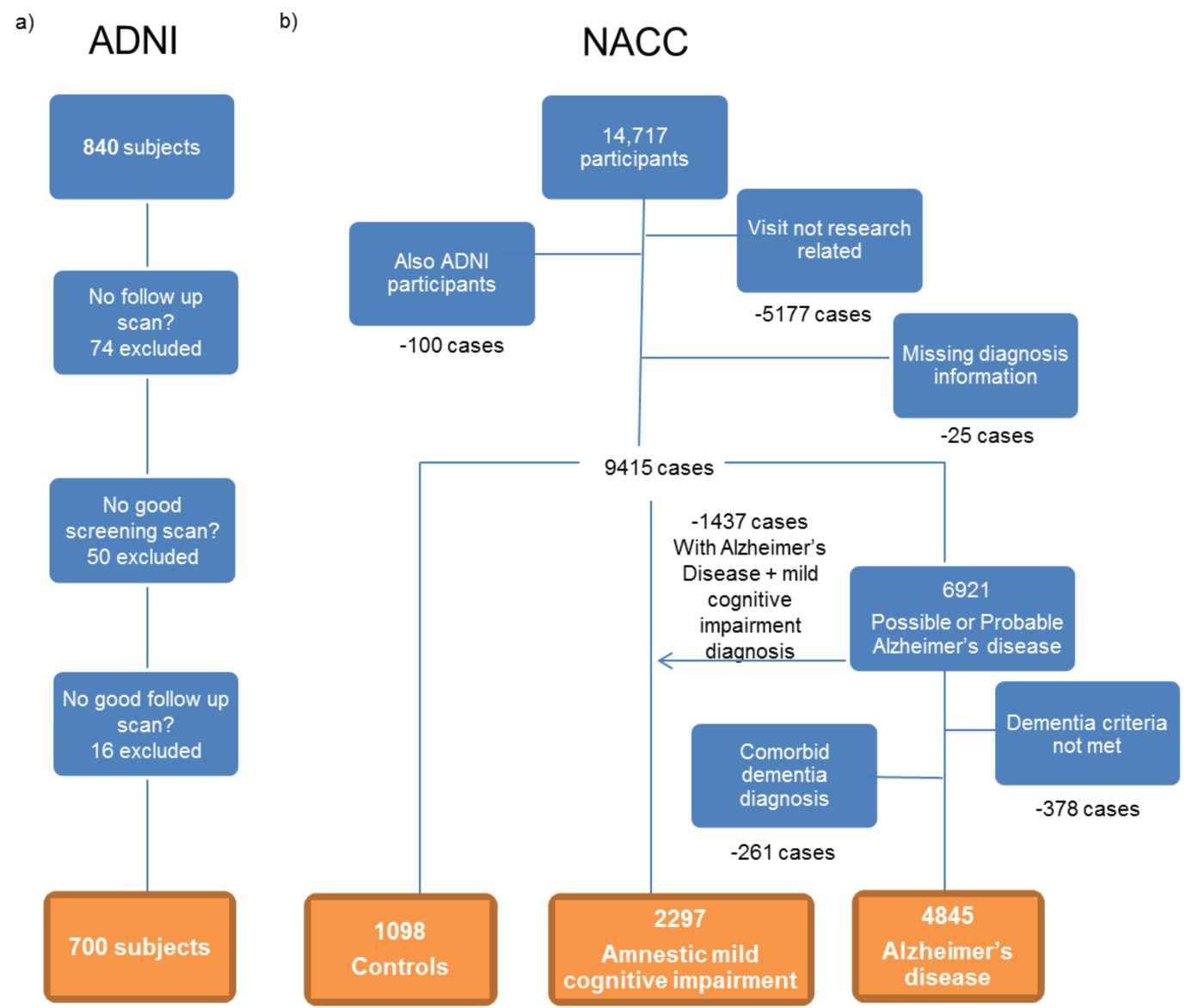
**Supplementary figure 1**: Flowchart showing the selection of subjects for analysis for (a) ADNI and (b) NACC. Abbreviations; ADNI (Alzheimer’s Disease Neuroimaging Initiative), NACC (National Alzheimer’s Co-ordinating Center).


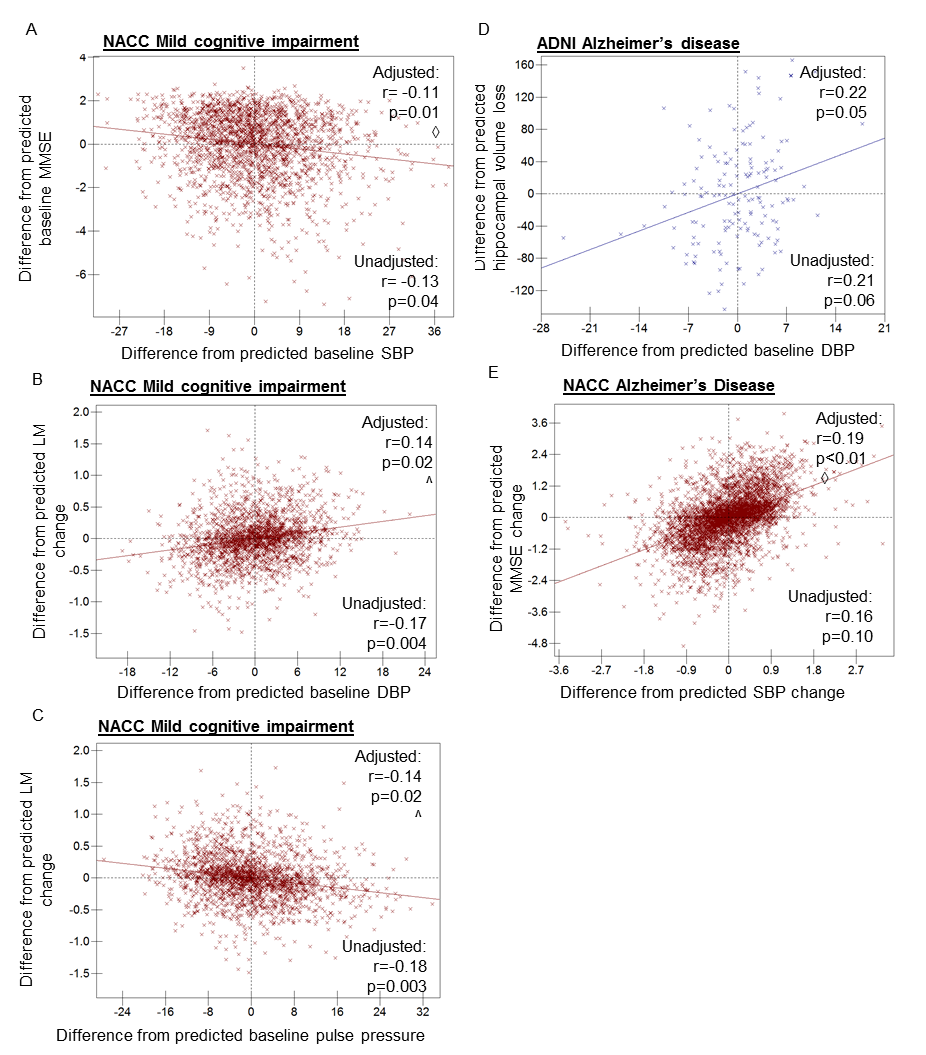


**Supplementary figure 2:** Graphs to demonstrate relationships between blood pressure (BP) and baseline cognition, subsequent cognitive or hippocampal change. Graphs of participant level residuals, demonstrating random effect correlations. Graphs a-d show correlations in NACC mild cognitive impairment patients, between (a) baseline systolic BP (SBP) and baseline MMSE; (b) baseline diastolic BP (DBP) and change in logical immediate story recall (LM); (c) baseline pulse pressure and change in LM, (d) baseline DBP and hippocampal volume in ADNI Alzheimer’s disease patients, (1e) change in SBP and change in MMSE in NACC Alzheimer’s disease patients. All ADNI relationships are corrected for APOE genotype, age, sex and TIV. All NACC relationships adjusted for age and sex. For scatterplots of residuals demonstrating whole-brain/hippocampal correlations with BP, MMSE was the third outcome in the model.

| Fixed effects | | ADNI | | | NACC | | |
| --- | --- | --- | --- | --- | --- | --- | --- |
|  |  | Control | Mild Cognitive Impairment | Alzheimer’s Disease | Control | Mild Cognitive Impairment | Alzheimer’s Disease |
| **Baseline** | Systolic (mmHg) | 132.53 [129.54, 135.52]  (<0.01) | 136.05, [133.14, 138.95] (<0.01) | 133.48 [129.33, 137.63] (<0.01) | 135.1 [133.8, 136.4]  (p<0.01) | 136.4 [135.37, 137.44] (p<0.01) | 135.8 [135.07, 136.54]  (p<0.01) |
|  | Diastolic (mmHg) | 73.78 [71.97, 75.59]  (<0.01) | 73.95 [72.28, 75.63]  (<0.01) | 73.94 [71.57, 76.31]  (<0.01) | 73.63 [72.9, 74.36]  (p<0.01) | 74.39 [73.82, 74.96]  (p<0.01) | 74.32 [73.92, 74.73] (p<0.01) |
|  | Pulse  (mmHg) | 58.82 [56.08, 61.55]  (<0.01) | 62.04 [59.54, 64.55]  (<0.01) | 59.5 [55.86, 63.15]  (<0.01) | 61.48 [60.37, 62.58]  (p<0.01) | 62.01 [61.13, 62.89]  (p<0.01) | 61.48 [60.85, 62.11] (p<0.01) |
| **Change** | Systolic (mmHg/yr.) | -0.11 [-1.11, 0.89]  (0.83) | -0.43 [-1.45, 0.59]  (0.41) | 0.25 [-1.46, 1.95]  (0.78) | -0.18 [-0.5, 0.14]  (0.28) | -0.34 [-0.71, 0.02]  (0.07) | -0.98 [-1.33, -0.64] (p<0.01) |
|  | Diastolic (mmHg/yr.) | -0.49 [-1.01, 0.04]  (0.07) | -0.87 [-1.40, -0.34]  (<0.01) | 0.75 [-0.19, 1.7]  (0.12) | -0.03 [-0.21, 0.15]  (0.72) | -0.35 [-0.56, -0.15] (p<0.01) | -0.41 [-0.61, -0.21] (p<0.01) |
|  | Pulse  (mmHg/yr.) | 0.36 [-0.49, 1.2]  (0.41) | 0.44 [-0.43, 1.32]  (0.32) | -0.5 [-2.11, 1.12]  (0.55) | -0.15 [-0.41, 0.12]  (0.28) | 0.02 [-0.31, 0.34]  (0.92) | -0.58 [-0.87, -0.28] (p<0.01) |
| **Baseline** | Brain volume (ml) | 1,120 [1,060 - 1,181]  (<0.01) | 1,046 [1,003 - 1,088] (<0.01) | 1,011 [937 - 1,086] (<0.01) | N/A | | |
|  | Hippocampal volume (ml) | 5.03 [4.3 - 5.8]  (<0.01) | 4.661 [4.055 - 5.266]  (<0.01) | 3.4 [2.348 - 4.506]  (<0.01) |  |  |  |
| **Change** | Brain volume (ml/yr.) | 6.23 [5.41, 7.04]  (<0.01) | 10.71 [9.38, 12.05]  (<0.01) | 14.15 [12.06, 16.24]  (<0.01) |  |  |  |
|  | Hippocampal volume (ml/yr.) | 0.06 [0.05, 0.08]  (<0.01) | 0.14 [0.12, 0.16]  (<0.01) | 0.17 [0.14, 0.21]  (<0.01) |  |  |  |
| **Baseline** | MMSE (/30) | 29.16 [28.98, 29.35]  (<0.01) | 26.99 [26.6, 27.38]  (<0.01) | 23.69 [23.03, 24.35]  (<0.01) | 28.79 [28.67, 28.91]  (p<0.01) | 27.04 [26.9, 27.19]  (p<0.01) | 19.52 [19.25, 19.79] (p<0.01) |
|  | DSST (/93) | 49.13 [47.01, 51.25] (<0.01) | 38.34 [35.83, 40.86] (<0.01) | 28.19 [24.03, 32.35] (<0.01) | 42.39 [41.55, 43.22]  (p<0.01) | 37.85 [37.17, 38.54]  (p<0.01) | 24.62 [23.96, 25.27] (p<0.01) |
|  | LM (/25) | 14.76 [14.06, 15.46] (<0.01) | 7.94 [7.3, 8.57]  (<0.01) | 4.24 [3.36, 5.13]  (<0.01) | 12.44 [12.14, 12.74]  (p<0.01) | 8.75 [8.52, 8.97]  (p<0.01) | 3.97 [3.82, 4.11]  (p<0.01) |
| **Change** | MMSE  (Δ/yr.) | 0.03 [-0.05, 0.12]  (0.43) | -0.83 [-1.14, -0.53]  (<0.01) | -2.47 [-3.29, -1.64]  (<0.01) | -0.52 [-0.58, -0.46]  (p<0.01) | -0.81 [-0.91, -0.72]  (p<0.01) | -1.21 [-1.56, -0.86] (p<0.01) |
|  | DSST (Δ/yr.) | 0.37 [-0.1, 0.85]  (0.13) | -1.43 [-2.19, -0.67]  (<0.01) | -4.05 [-1.44, -2.56]  0.76 | -0.18 [-0.5, 0.14]  0.28 | -1.8 [-2.02, -1.58]  (p<0.01) | -3.11 [-3.43, -2.79] (p<0.01) |
|  | LM (Δ/yr.) | 0.3 [0.04, 0.55]  (0.03) | -0.12 [-0.41, 0.18]  (0.45) | -0.76 [-1.18, -0.34]  (<0.01) | -0.46 [-0.54, -0.39]  (p<0.01) | -0.39 [-0.47, -0.31]  (p<0.01) | -0.56 [-0.62, -0.5]  (p<0.01) |

**Supplementary table 1:** Fixed effects from mixed models assessing the relationship between blood pressure (systolic, diastolic and pulse pressure), cognition ((mini-mental state examination (MMSE), logical memory immediate story recall (LM) and WAIS-R Digit Symbol (DSST)), and whole-brain or hippocampal volume change. Estimates are shown with (p values) and [95% confidence intervals]. For brevity we do not report fixed effects (FE) from each model, which can be very similar between models. In ADNI all BP fixed effects values are taken from models where DSST and whole-brain volume change are the second and third outcomes. Whole-brain and hippocampal volume change estimates are taken from models where DSST and SBP are the second and third outcomes. All neuropsychology fixed effects are from models with whole-brain volume change and SBP as the second and third outcomes. Baseline volumes for the brain and hippocampus are from linear regression. In NACC all BP fixed effects are taken from models where DSST is the second outcome. All neuropsychology fixed effects are taken from models where SBP is the second outcome. ADNI models are adjusted for age, gender, intracranial volume and APOE e4 status, NACC models are adjusted for age and gender.

|  | **SBP** | | | **DBP** | | | **PP** | | |
| --- | --- | --- | --- | --- | --- | --- | --- | --- | --- |
|  | **Controls** | **Mild Cognitive Impairment** | **Alzheimer’s Disease** | **Controls** | **Mild Cognitive Impairment** | **Alzheimer’s Disease** | **Controls** | **Mild Cognitive Impairment** | **Alzheimer’s Disease** |
| Whole-brain volume | 1,120  [1,060, 1,181] | 1,046  [1,003, 1,088] | 1,011  [936, 1,086] | 1,132  [1,077, 1,186] | 1,064  [1,020, 1,109] | 1,011  [937, 1,086] | 1,070  [1,040, 1,101] | 1,053  [1,030, 1,076] | 1,013  [972, 1,053] |
| BP effect on whole-brain volume | -0.41  [-0.86, 0.03]  (0.07) | 0.13  [-0.18, 0.44]  (0.40) | -0.01  [-0.55, 0.52]  (0.96) | -0.88  (0.01)  [-1.59,  -0.17] | -0.01  (0.96)  [-0.60, 0.58] | -0.01  (0.96)  [-0.55, 0.52] | -0.08  (0.74)  [-0.57, -0.40] | 0.18  (0.33)  [-0.18, 0.53] | -0.05  (0.86)  [-0.63, 0.53] |
| Hippocampal volume | 5.03  [4.29, 5.77] | 4.66  [4.05, 5.27] | 3.43  [2.35, 4.51] | 5.47  [4.80, 6.14] | 4.83  [4.20 , 5.47] | 3.69  [2.66, 4.72] | 4.96  [4.59, - 5.33] | 4.59  [4.26, 4.92] | 3.88  [3.29, 4.47] |
| BP effect on hippocampal volume | 0.00  [-0.00, 0.01]  (0.77) | -0.00  [-0.00, 0.00]  (0.92) | 0.01  [-0.00, 0.01]  (0.20) | -0.00  [-0.01, 0.00]  (0.31) | -0.00  [-0.01 , 0.01]  (0.53) | 0.01  [-0.01, 0.02]  (0.41) | 0.00  [-0.00 , 0.01]  (0.32) | 0.00  [-0.00, 0.01]  (0.79) | 0.00  [-0.00, 0.01]  (0.39) |

**Supplementary table 2:** Results of the regression model assessing the relationship between whole-brain volume and blood pressure, and hippocampal volume and blood pressure measure (systolic (SBP), diastolic (DBP) or pulse pressure (PP)) in control (C), MCI and AD patients. Estimates are shown for difference in whole-brain or hippocampal volume (ml) with (p values) and [95% confidence intervals] for a 1 mmHg increase in blood pressure measure. Models were run separately in each diagnostic group and adjusted for APOE genotype (binary variable indicating presence of an e4 allele), age, gender and total intracranial volume.

| ADNI Blood pressure and brain atrophy rate | Controls | Mild Cognitive Impairment | Alzheimer’s disease | |
| --- | --- | --- | --- | --- |
| BP variable | Correlation with whole-brain volume change | | | |
| Baseline SBP | 0.02  (0.87) | 0.11  (0.12) | | 0.09  (0.47) |
| Baseline DBP | -0.18  (0.11) | 0.10  (0.18) | | 0.12  (0.34) |
| Baseline PP | 0.13  (0.22) | 0.07  (0.33) | | 0.09  (0.52) |
| Change in SBP | 0.09  (0.53) | -0.17  (0.17) | | Inestimable |
| Change in DBP | 0.43  (0.07) | -0.17  (0.28) | | Inestimable |
| Change in PP | -0.07  (0.64) | -0.13  (0.29) | | -0.45  (0.24) |

**Supplementary table 3**: Systolic blood pressure (SBP), diastolic blood pressure (DBP), and pulse pressure (PP)) and brain volume change are correlated in Alzheimer’s disease Neuroimaging Initiative 1 (ADNI1). Random effects correlations (r and (p values)) are shown. Analyses are adjusted for sex, age, APOE ε4 genotype and total intracranial volume.

| ADNI Neuropsychology | | Control | | | Mild Cognitive Impairment | | | Alzheimer’s disease | | |
| --- | --- | --- | --- | --- | --- | --- | --- | --- | --- | --- |
| Correlation of interest | BP  variable | Correlation with neuropsychology | | | | | | | | |
|  |  | MMSE | LM | DSST | MMSE | LM | DSST | MMSE | LM | DSST |
| Baseline BP measure, Baseline neuropsychology | SBP | -0.11  (0.37) | -0.18  (0.06) | -0.01  (0.87) | 0.02  (0.80) | -0.08  (0.33) | -0.03  (0.71) | -0.12  (0.37) | -0.07  (0.54) | -0.06  (0.56) |
|  | DBP | -0.24  (0.05) | -0.05  (0.47) | 0.08  (0.34) | -0.06  (0.50) | -0.03  (0.73) | -0.06  (0.39) | -0.06  (0.65) | 0.11  (0.30) | -0.07  (0.51) |
|  | PP | 0.03  (0.79) | -0.16  (0.09) | -0.07  (0.45) | 0.07  (0.43) | -0.07  (0.39) | 0.01  (0.91) | -0.12  (0.45) | -0.16  (0.20) | -0.07  (0.56) |
| Baseline BP measure, Change in neuropsychology | SBP | -0.05  (0.81) | -0.06  (0.71) | -0.28  (0.36) | -0.06  (0.41) | -0.01  (0.90) | -0.01  (0.89) | -0.06  (0.62) | 0.04  (0.84) | 0.10  (0.53) |
|  | DBP | 0.05  (0.83) | -0.14  (0.30) | -0.25  (0.40) | 0.02  (0.78) | 0.09  (0.35) | -0.03  (0.74) | -0.07  (0.55) | -0.19  (0.36) | -0.02  (0.92) |
|  | PP | -0.05  (0.78) | 0.03  (0.86) | -0.14  (0.64) | -0.09  (0.22) | -0.08  (0.41) | 0.01  (0.94) | -0.07  (0.57) | 0.09  (0.65) | 0.09  (0.61) |
| Change in BP measure, Change in neuropsychology | SBP | 0.12  (0.67) | -0.11  (0.61) | -0.76  (0.08) | 0.16  (0.21) | 0.09  (0.58) | 0.14  (0.33) | Inestim-able | Inestim-able | Inestim-able |
|  | DBP | -0.55  (0.28) | -0.44  (0.23) | -0.51  (0.45) | -0.06  (0.73) | -0.19  (0.34) | 0.12  (0.53) | Inestim-able | Inestim-able | Inestim-able |
|  | PP | 0.31  (0.29) | 0.04  (0.85) | -0.74  (0.12) | 0.22  (0.08) | 0.20  (0.20) | 0.11  (0.45) | 0.39  (0.27) | 0.43  (0.49) | 0.17  (0.69) |

**Supplementary Table 4:** Table showing correlations between blood pressure (BP) measures (systolic (SBP), diastolic (DBP and PP) and neuropsychology in the ADNI dataset Random effects correlations (correlation (r) and p values) are shown. BP and neuropsychology were jointly modelled as outcomes for each type of BP measurement (SBP, DBP and PP) and for each cognitive test (MMSE (Mini-mental state examination), LM (logical immediate story recall), and (DSST (WAIS-R Digit Symbol)). Whole-brain volume change was the third outcome. Analyses are adjusted for sex, age, APOE e4 genotype (binary variable indicating presence of an e4 allele) and total intracranial volume.

| **NACC with APOE adjustment** | | **Control** | | | **Mild Cognitive Impairment** | | | **Alzheimer’s disease** | | |
| --- | --- | --- | --- | --- | --- | --- | --- | --- | --- | --- |
|  |  | MMSE | LM | DSST | MMSE | LM | DSST | MMSE | LM | DSST |
| Baseline BP, Baseline neuropsychology | SBP | -0.04  (0.63) | -0.09  (0.1) | -0.07  (0.17) | -0.13  (0.01) | -0.11  (0.01) | -0.04  (0.27) | 0.10  (0.001) | 0.00  (>0.9) | 0.03  (0.38) |
|  | DBP | 0.08  (0.35) | 0.00  (0.95) | 0.06  (0.23) | -0.07  (0.14) | -0.07  (0.13) | -0.06  (0.19) | 0.05  (0.11) | -0.02  (0.61) | 0.00  (>0.9) |
|  | PP | -0.11  (0.23) | -0.1  (0.06) | -0.12  (0.02) | -0.11  (0.03) | -0.09  (0.05) | -0.02  (0.66) | 0.09  (0.003) | 0.01  (0.8) | 0.03  (0.34) |
| Baseline BP, Change in neuropsychology | SBP | 0.00  (0.97) | 0.00  (>0.9) | 0.02  (0.76) | -0.12  (0.02) | -0.07  (0.32) | -0.10  (0.08) | 0.02  (0.65) | 0.09  (0.2) | 0.01  (0.87) |
|  | DBP | -0.04  (0.47) | -0.05  (0.47) | -0.09  (0.18) | 0.06  (0.26) | 0.12  (0.07) | 0.02  (0.73) | -0.02  (0.61) | 0.05  (0.46) | -0.04  (0.51) |
|  | PP | 0.03  (0.59) | 0.04  (0.61) | 0.09  (0.21) | -0.18  (0.001) | -0.16  (0.02) | -0.14  (0.03) | 0.04  (0.39) | 0.07  (0.3) | 0.05  (0.37) |
| Change in BP, Change in neuropsychology | SBP | 0.08  (0.5) | 0.05  (0.65) | 0.01  (0.91) | 0.37  (0.001) | 0.29  (0.01) | 0.43  (0.00003) | 0.22  (0.01) | -0.01  (0.94) | 0.09  (0.52) |
|  | DBP | 0.11  (0.34) | 0.04  (0.75) | 0.08  (0.51) | 0.03  (0.8) | -0.02  (0.87) | 0.17  (0.12) | 0.14  (0.37) | -0.10  (0.59) | -0.04  (0.76) |
|  | PP | 0.02  (0.23) | 0.03  (0.82) | -0.04  (0.78) | 0.43  (0.00004) | 0.36  (0.002) | 0.40  (0.0001) | 0.20  (0.01) | 0.06  (0.76) | 0.09  (0.54) |

**Supplementary table 5**: Table showing correlations between blood pressure (BP) and neuropsychology in the NACC dataset. Random effects correlations (correlation (r) and p values) are shown. BP and neuropsychology were jointly modelled as outcomes for each type of BP measurement (systolic (SBP), diastolic (DBP) and pulse pressure (PP)), and for each neuropsychology test (MMSE (Mini-mental state examination), LM (logical immediate story recall) and DSST (WAIS-R Digit Symbol)). Analyses are adjusted for sex, age and APOE e genotype (binary variable indicating presence of an e4 allele).

| NACC with antihypertensive adjustment | | Control | | | Mild Cognitive Impairment | | | Alzheimer’s Disease | | |
| --- | --- | --- | --- | --- | --- | --- | --- | --- | --- | --- |
|  |  | MMSE | LM | DSST | MMSE | LM | DSST | MMSE | LM | DSST |
| Baseline BP, Baseline neuropsychology | SBP | -0.08 (0.25) | -0.12 (0.01) | -0.06 (0.17) | -0.12  (0.005) | -0.09 (0.01) | -0.03 (0.35) | 0.08 (0.001) | 0.01 (0.73) | 0.00 (0.96) |
|  | DBP | 0.00 (0.96) | -0.06 (0.22) | 0.04 (0.37) | -0.05 (0.24) | -0.04 (0.34) | -0.06 (0.19) | 0.03 (0.27) | -0.03 (0.29) | -0.04 (0.19) |
|  | PP | -0.10 (0.16) | -0.10 (0.04) | -0.10 (0.03) | -0.11 (0.01) | -0.09 (0.02) | -0.03 (0.35) | 0.08 (0.002) | 0.03 (0.29) | 0.02 (0.46) |
| Baseline BP, Change in neuropsychology | SBP | -0.04 (0.44) | -0.03 (0.62) | -0.02 (0.79) | -0.08 (0.08) | -0.05 (0.42) | -0.13 (0.01) | -0.01 (0.76) | 0.00 (0.95) | -0.04 (0.44) |
|  | DBP | -0.04 (0.42) | -0.06 (0.33) | -0.07 (0.29) | 0.05 (0.25) | 0.14 (0.02) | 0.03 (0.58) | -0.04 (0.35) | 0.04 (0.53) | -0.02 (0.7) |
|  | PP | -0.02 (0.79) | 0.01 (0.93) | 0.13 (0.67) | -0.13 (0.004) | -0.16 (0.01) | -0.17 (0.001) | 0.01 (0.84) | -0.02 (0.75) | -0.02 (0.69) |
| Change in BP, Change in neuropsychology | SBP | 0.09 (0.37) | 0.02 (0.88) | 0.01 (0.96) | 0.27 (0.001) | 0.20 (0.06) | 0.43 (0.00001) | 0.18 (0.06) | 0.06 (0.64) | 0.08 (0.54) |
|  | DBP | 0.06 (0.54) | 0.03 (0.76) | -0.02 (0.85) | 0.05 (0.61) | -0.04 (0.72) | 0.17 (0.12) | 0.15 (0.07) | -0.04 (0.7) | -0.04 (0.76) |
|  | PP | 0.06 (0.67) | -0.01 (0.92) | 0.02 (0.88) | 0.30 (0.001) | 0.27 (0.01) | 0.40 (0.00003) | 0.13 (0.25) | 0.11 (0.43) | 0.09 (0.54) |

**Supplementary table 6**: Table showing correlations between blood pressure (BP) and neuropsychology in the NACC dataset. Random effects correlations (correlation (r) and p values) are shown. BP and neuropsychology were jointly modelled as outcomes for each type of BP measurement (systolic (SBP), diastolic (DBP) and pulse pressure (PP)), and for each neuropsychology test (MMSE (Mini-mental state examination), LM (logical immediate story recall) and DSST (WAIS-R Digit Symbol)). Analyses are adjusted for sex, age and antihypertensive treatment (binary variable indicating whether participants were taking BP lowering medication at each timepoint).

| NACC | Hypotensive | | Normotensive | | Hypertensive | |
| --- | --- | --- | --- | --- | --- | --- |
|  | **Non-medicated** | **Medicated** | **Non-medicated** | **Medicated** | **Non-medicated** | **Medicated** |
|  | 1. Non-medicated Hypotensive | 2.Medicated Hypotensive | 3. Non-medicated normotensive | 4. Medicated normotensive | 5. Non-medicated  hypertensive | 6. Medicated hypertensive |
| n | 103 | 156 | 1168 | 2084 | 928 | 374 |
| Age (SD) range | 77.11 (11.2)  47, 100 | 80.7 (7.3)  59, 97 | 71.83 (11.06)  36, 99 | 77.27 (8.5)  41, 102 | 74.33 (10.36)  40, 104 | 76.9 (10.15)  50, 110 |
| APOE status (% e4 carriers) | 59 | 53 | 59 | 59 | 61 | 59 |
| Sex  (% male) | 32 | 53 | 46 | 49 | 41 | 52 |
| Education (SD) | 14.07 (4.1) | 14.51 (10.6) | 15.40 (9.0) | 14.47 (7.71) | 15.14 (9.1) | 14.98 (10.5) |
| Baseline SBP | 119.78  [115.71, 123.84] | 121  [115.97, 126.03] | 123.46  [122.52, 124.4] | 137.12  [136.05, 138.19] | 148.96  [147.60, 150.32] | 158.76  [155.30, 162.22] |
| Change in SBP | 3.22  [0.42, 6.02]  (0.02) | 2.4  [-0.5, 5.3]  (0.1) | 0.89  [0.21, 1.57]  (0.01) | -0.9  [-1.46, -0.35]  (0.001) | -4.76  [-5.58, -3.94]  (<0.001) | -6.32  [-8.06, -4.58]  (<0.001) |
| Baseline MMSE | 19.56  [17.5, 21.61] | 19.98  [18, 21.97] | 19.11  [18.51, 19.71] | 20.03  [19.62, 20.43] | 19.05  [18.47, 19.63] | 19.40  [18.38, 20.43] |
| Change in MMSE | -2.37  [-2.9, -1.84]  (<0.001) | -2.67  [-3.62, -1.72]  (<0.001) | -2.38  [-2.64, -2.13]  (p<0.001) | -2.02  [-2.21, -1.83]  (<0.001) | -2.48  [-2.73, -2.22]  (p<0.001) | -2.46  [-2.91,-2.01]  (p<0.001) |

**Supplementary table 7:** Results for AD patients split by baseline hypertensive status. Demographics, and fixed effects, resulting from joint models of systolic blood pressure (SBP) and mini-mental state examination (MMSE) change in AD patients by hypertensive status. Demographics are given as mean with SD, unless specified. Fixed effects (baseline SBP and baseline MMSE, change in SBP, change in MMSE) are given as estimate, [95% Confidence interval] (p value). Patients were split by hypertensive status (based on baseline blood pressure reading and baseline antihypertensive usage). Analyses are adjusted for sex and age.
